# Functional Outcome and Independence Among In‐Hospital Cardiac Arrest Patients in South India: A Prospective Longitudinal Study

**DOI:** 10.1155/nrp/6036368

**Published:** 2026-04-24

**Authors:** Anandhi Deva Amirtharaj, Malarvizhi Suresh

**Affiliations:** ^1^ Department of Adult and Critical Care Nursing, College of Nursing, Sultan Qaboos University, Muscat, Oman, squ.edu.om; ^2^ College of Nursing, P.I.M.S, Affiliated to Pondicherry University, Kanagachettikulam, Pondicherry, 605014, India

**Keywords:** activities of daily living, cardiac arrest, cardiopulmonary resuscitation, critical care outcomes, level of independence, reliability, return of spontaneous circulation, validity

## Abstract

**Introduction:**

The duration of cardiopulmonary resuscitation (CPR) has a strong effect on the prognosis and recovery of the patients. However, there is no standardization or Core Outcome Set for In‐Hospital Cardiac Arrest. The main aim of the study is to assess the impact of CPR duration on functional outcome, survival, and the level of independence among cardiac arrest patients. Methods: For this observational study, 121 patients were selected from two tertiary hospitals in South India. Data were collected from patients during the event of cardiac arrest with functional outcome and level of independence measured immediate post‐CPR, Day 30, and Day 90 using standardized tools, namely, cerebral performance category and Katz index tool for the level of independence

**Results:**

The mean and median age was 62.49 ± 13.18 and 65 years (IQR, 54–73 years), respectively, with 89 (73.6%) male patients. At the end of CPR, 55.3% of patients achieved ROSC and with a mean and median CPR duration of 18.97 ± 14.7 min and 15 min (IQR, 10 and 26 min), respectively. Overall mortality rates were 33.8% immediately following CPR, 56.1% by Day 30, and 58.67% by Day 90, with an overall survival rate of 41.3% at 90 days. Immediately post‐CPR, 95% of patients were in an unfavorable neurological state; this proportion improved to 37.19% by Day 30, with no significant further improvement observed by Day 90. Level of independence demonstrates that 83.7% of the patients were dependent during the immediate post‐CPR period with improvement to partial independence among 84.9% of patients by Day 30. The mean score of favorable and unfavorable outcome on Day 90 was 1.02 and 4.93, respectively. The mean score of dependency and partially dependent on Day 90 among the survivor was 0 and 4.78, respectively. The predictors are ROSC, use of life‐saving drugs, and CPR duration.

**Conclusion:**

Utstein reporting system must be integrated for cardiac arrest patients. Cardiac resuscitation and termination must be based on established policy and guidelines to maintain neurological integrity to sustain the quality of life during the post‐CPR.

## 1. Introduction

Cardiac arrest (CA) is the leading noncommunicable disease (NCD) responsible for almost 75% of deaths, and WHO in their 2023 report states the alarming fact projecting an increase to 86% by 2048, while the United Nations projects the total global annual death to 90 million by 2048 of which 77 million deaths will be due to NCD [1].

CA, the most common preceding sign to death, is caused by various manifestations defined as the absolute stoppage of cardiac functioning, which leads to the absence of circulation, and these are classified as in‐hospital CA (IHCA) and out‐hospital CA (OHCA), which is reported as a universal problem [2, 3]. The incidence of IHCA globally is reported as 1.7 per 1000 hospital in Sweden, 0.15% in China, and 0.67 per 1000 in Australia, while OHCA in Europe and the United States is documented as 2.7 million and 3.8 million, respectively [2].

There is a significant gap in the accurate documentation and reporting of CA in South Asian countries [4]. A systematic review published in 2020 estimated that approximately 5.5 million cases of CA occur annually in South Asia [5]. However, in India, there is no comprehensive national registry to accurately record and monitor the incidence and outcomes of CA. Consequently, the available estimates are likely to represent only the tip of the iceberg, underestimating the true burden of the disease. With the projected rise in cardiovascular morbidity and mortality, the incidence and prevalence of CA are expected to increase further.

India lacks a coordinated emergency medical system and a standardized Utstein‐style registry for CA reporting [6]. Evidence from South and Southeast Asia indicates a high incidence of CA with comparatively low survival rates. Although resuscitation practices follow American Heart Association (AHA) guidelines, inconsistencies in the termination of resuscitation (ToR) practices persist due to limited regional research and awareness [7]. Most existing studies focus on survival and immediate neurological outcomes, with limited attention to holistic recovery among survivors. Level of independence (LOI), an important indicator of functional recovery and quality of life after cardiopulmonary resuscitation (CPR), remains underexplored in Asia. Functional outcome, survival, and quality of life are recognized core outcome measures in CA research; however, these outcomes are insufficiently studied in India.

Therefore, this study aims to determine the median duration of CPR and evaluate its impact on the neurological outcome and LOI among patients experiencing CA.

## 2. Methodology

A quantitative, nonexperimental, descriptive analytical longitudinal design was employed to examine the relationship between CPR duration and subsequent functional independence among IHCA patients. The independent variable was CPR duration (measured in minutes from initiation to return of spontaneous circulation [ROCS] or termination), while the dependent variables were functional outcome and LOI, assessed through neurological recovery, cognitive status, and ability to perform activities of daily living (ADL). The study was conducted from March 2022 to February 2023 at two tertiary care hospitals in South India—Pondicherry Institute of Medical Sciences and Madras Medical Mission—with outcomes evaluated immediately post‐CPR, at 30 days, and at 90 days. Adult IHCA patients (≥ 18 years) were included, while those with advance directives, confirmed COVID‐19 status, or declared dead on arrival were excluded. Using Cochran’s formula with a finite population of 150 cases (average admission over the previous year), 95% confidence level (*Z* = 1.96), and a 0.05 margin of error, the initial sample size was 98; after adjusting for a 20% anticipated attrition rate, the final sample size was 121 participants [8]. A consecutive sampling technique was used to recruit all eligible IHCA patients who met the inclusion criteria until the required sample size was met.

## 3. Description of the Tool

### 3.1. Study Instruments

The following scales were used to measure the functional outcome and LOI using the standardized tools, namely, the cerebral performance category (CPC) and LOI by Katz.

Data were collected using two instruments. Tool 1 was self‐structured Performa divided into three sections: (A) sociodemographic characteristics (age, gender, origin, marital status), (B) clinical variables related to the CA event based on the Utstein style [9] (e.g., initial rhythm, witnessed status, time to resuscitation, medications, defibrillation, ROSC), and (C) the total duration of CPR. The reliability of this self‐prepared tool was established through the test–retest method (*r* = 0.80).

Tool 2 measured patient outcomes using two standardized scales. The Glasgow–Pittsburgh CPC scale was used to assess neurological outcomes, with scores ranging from 1 (Good Cerebral Performance) to 5 (Brain Death). A CPC score of 1 or 2 was classified as a favorable outcome, while scores of 3–5 were considered unfavorable [10, 11]. The Katz index of independence in ADL was employed to evaluate functional status. This six‐item scale assesses independence in bathing, dressing, toileting, transferring, continence, and feeding. Total scores classify patients as Dependent (0–2), Partially Dependent (3–5), or Independent (6) [12].

### 3.2. Reliability and Validity of the Tool

Both standardized tools demonstrated strong psychometric properties in this study. The CPC scale showed a validity index of 0.78 and high reliability for favorable (0.96) and unfavorable (0.82) outcomes [13]. The Katz index exhibited excellent internal consistency (*α* = 0.87–0.94), a validity index of 0.838, and a test–retest reliability of 0.999 [14–16]. The validity and reliability of the two standardized tools in this study were assessed using Cronbach’s α score for functional outcome and LOI is 0.891 and 0.827, respectively.

### 3.3. Ethical Considerations

The ethical permission (IRB‐PIMS/PhD(N)/20/14 dated May 13, 2021) and MMM (MMM/PhD/22/4 dated April 04, 2022) were obtained prior to the gathering of data. Participation in the study was granted by the caretaker or family member, which was entirely voluntary, and participants were informed of their right to withdraw at any stage without any penalty or impact on their care. Strict measures were implemented to ensure confidentiality, and no identifying information of participants or their significant others was recorded. All collected data were securely stored in a password‐protected file accessible only to the investigators.

### 3.4. Data Collection Process

Data collection was initiated upon activation of the CODE BLUE system, indicating the occurrence of an IHCA. Eligible participants were identified based on inclusion criteria observed with documents and level of care, and informed consent was obtained from the immediate family member at an appropriate time, considering the sensitive nature of the event. Participant privacy and confidentiality were maintained through a coding system in which all personal identifiers were replaced with unique study codes.

Baseline data were collected immediately after the post‐CPR period. Subsequent assessments were conducted at Day 30 or at discharge, whichever occurred earlier. For patients who remained hospitalized, evaluations were completed on‐site, whereas those discharged were followed up at 90 days through telephone interviews or scheduled outpatient visits, based on feasibility. Each assessment utilized standardized research instruments and required approximately 10–15 min per participant.

### 3.5. Data Analysis and Interpretation

Data were analyzed using IBM SPSS Statistics (Armonk, NY: IBM Corp.). Both descriptive and inferential statistics were applied to examine patterns, correlations, and associations. Continuous variables were summarized using mean and standard deviation, while categorical variables were presented as frequencies and percentages.

Karl–Pearson correlation coefficients were used to assess the relationships between continuous variables, and the Fisher exact test or chi‐square test was applied for categorical variables, as appropriate. Repeated‐measures ANOVA was performed to evaluate the changes in outcomes across time points, and paired *t* tests were used to compare the mean differences over the observed intervals. Univariate regression analysis was conducted to identify predictors of the primary outcome. A *p* value of < 0.05 was considered statistically significant for all analyses.

## 4. Results

### 4.1. Section 1.1: Description of Demographic Variables of the Participants

The sociodemographic characteristics of 121 participants were analyzed. The majority were male (*n* = 89, 73.6%). Participants’ ages ranged from 28 to 91 years, with a mean age of 62.49 ± 13.18 years and a median of 65 years (IQR: 54–73). Most participants (*n* = 73, 60.4%) were aged ≥ 61 years, consistent with the established association between advancing age and increased cardiac risk. The majority were married (*n* = 116, 95.8%). A substantial proportion resided in urban areas (*n* = 110, 90.9%), likely reflecting the urban location of one participating tertiary care centers.

### 4.2. Section 1.2: Description of Clinical Variables of the Participants

Section 1.2 describes the frequency and percentage of the clinical variables.

Among the 121 participants, 80 (66.1%) had no previously diagnosed comorbidities, indicating that CA may have been the first clinical presentation of an underlying disease. A primary cardiac cause was identified in 90 participants (74.4%), suggesting that many cardiac conditions remained undetected until the occurrence of the arrest. Secondary diagnoses were mainly respiratory and neurological disorders, which are part of the broader spectrum of NCDs and reflect their growing health burden. Of the total cohort, 94 participants (77.7%) were admitted to the intensive care unit (ICU), allowing for timely clinical management. CA was witnessed in 119 cases, and CPR was initiated by healthcare professionals in 117 cases. In 115 patients (95%), CPR was started within five minutes of arrest, which may be attributed to the in‐hospital acute care setting and the immediate availability of trained medical personnel.

**FIGURE 1:**
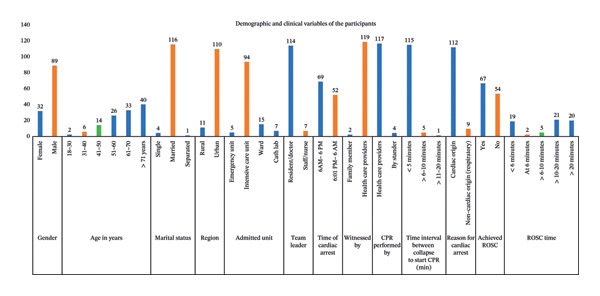
Frequency of sociodemographic variables and clinical variables of the participants.

Patient location and admission unit were associated with timely resuscitation. Most admissions were due to cardiac causes (*n* = 90, 74.4%), consistent with the finding that 112 arrests (92.6%) were cardiac in origin, while nine cases (7.4%) were respiratory. ROSC was achieved in 67 patients (55.3%). Among them, 21 patients (31.3%) achieved ROSC within 10–20 min of CPR, while 19 (28.3%) and two patients (2.9%) achieved ROSC in less than six minutes and at six minutes, respectively. Demographic and clinical characteristics are shown in Figure 1.

### 4.3. Section 1.3: Description of Duration of CPR Among the Participants

**FIGURE 2:**
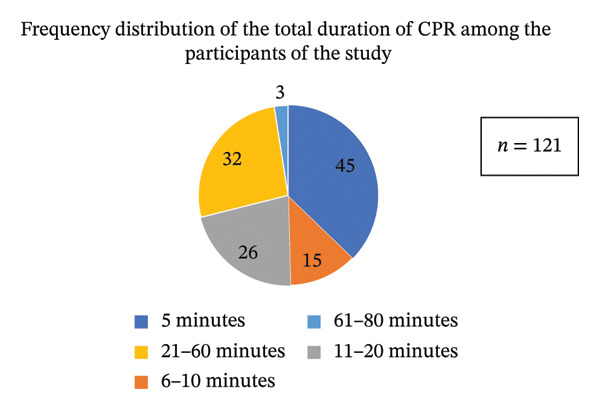
Frequency distribution of CPR duration among patients with cardiac arrest.

The mean CPR duration was 18.97 ± 14.7 min, with a median of 15 min (IQR: 10–26 min), ranging from 2 to 80 min. As shown in Figure 2, 45 patients received CPR for less than 5 min, and 15 patients underwent resuscitation for less than 10 min. CPR duration was 11–20 min in 26 patients and more than 20 min in 35 patients. Six patients required prolonged resuscitation exceeding 40 min, including two younger patients aged 41 and 34 years, while four patients experienced recurrent CA episodes that were documented as a single resuscitation event.

### 4.4. Description of the Survivors During the Period of Data Collection

**FIGURE 3:**
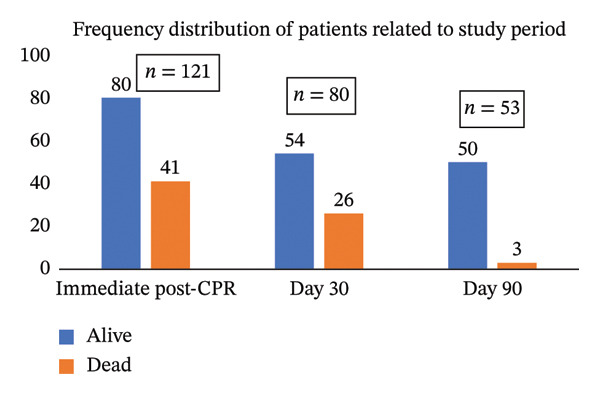
Frequency distribution of mortality and morbidity status of the cardiac arrest patients during the immediate post‐CPR, Day 30, and Day 90.

Of the 121 patients enrolled, 41 (33.8%) died immediately following CPR, while 80 patients survived the initial postresuscitation period. By Day 30, an additional 27 patients (33.75% of the survivors) died due to complications, leaving 53 survivors. Between Day 30 and Day 90, three more deaths (5.6%) were recorded, resulting in 50 survivors at the end of the follow‐up period. Overall, 71 patients (58.6%) died within 90 days, while 50 patients (41.3%) survived, as displayed in Figure 3. Mortality was highest immediately after CPR and decreased substantially after the first month of follow‐up.

### 4.5. Section 2: Description of Participants Based on Functional Outcome Dimensions in Terms of Favorable and Unfavorable Outcomes Among CA Patients

The distribution of functional outcomes demonstrated a progressive improvement in favorable outcomes over the follow‐up period. Immediately after CPR, only six (5%) patients had favorable outcomes, while 115(95%) patients had unfavorable outcomes with 80 survivors. By Day 30, favorable outcomes increased to 44 (56.19%) among 53 survivors, whereas unfavorable outcomes declined to 36. However, a minimal change was observed between Day 30 and Day 90, with 45 patients maintaining a favorable status and five patients remaining in the unfavorable category, indicating a plateau in functional recovery. The mean functional outcome scores showed a declining trend in the favorable group, decreasing from 1.5 immediately post‐CPR to 1.02 by Day 90, reflecting sustained functional recovery. In contrast, the unfavorable group exhibited consistently higher mean scores, increasing from 3.83 to 4.93 during follow‐up, indicating persistent severe disability. The overall mean functional outcome score was 3.72 ± 1.04 immediately post‐CPR, 3.48 ± 1.85 at Day 30, and 3.48 ± 1.91 at Day 90, with median scores ranging between 3 and 5, suggesting that most patients remained in moderate to severe disability range throughout the observation period.

### 4.6. Section 3: Description of Participants Based on the Dimension of LOI in Terms of Dependent, Partially Dependent, and Independent Among Cardiac Arrest Patients

**FIGURE 4:**
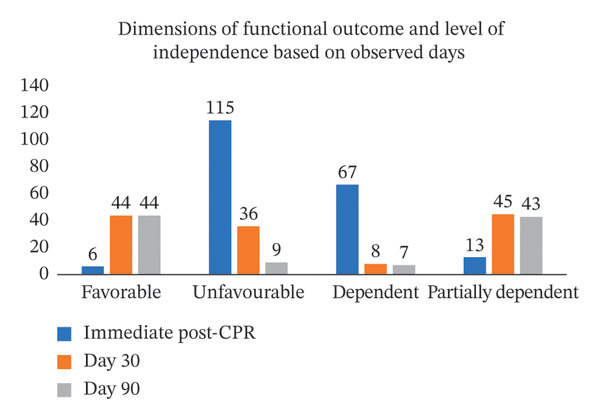
Frequency distribution of outcome of the patients and independence among survivors during immediate post‐CPR, Day 30, and Day 90.

The scoring classification of LOI as displayed in Figure 4 demonstrated that functional status improved progressively over time. Immediately after CPR, among the 80 survivors, 67 patients (83.75%) were classified as dependent, while 13 patients (16.25%) were partially dependent, indicating a markedly poor functional status in the immediate postresuscitation period.

By Day 30, a marked improvement in functional status was observed among 53 survivors. Dependency decreased substantially to eight patients (15.09%), while the majority of 45 patients (84.91%) were partially dependent. Although no patient achieved full independence at this stage, recovery in functional capacity was evident when compared to the immediate post‐CPR period.

Further improvement was noted by Day 90 among the 50 survivors, where functional status showed stabilization and sustained recovery. Dependency was minimal with seven patients (14%), while 43 patients (86%) were classified as partially dependent. None of the patients attained full independence; however, long‐term functional recovery was maintained among survivors.

The analysis of mean independence scores to the CPR duration across observation periods revealed a gradual increase in overall functional ability, with the mean score improving from 2.53 immediate post‐CPR to 3.81 on Day 30 and 4.30 on Day 90. The decreasing standard deviation over time indicated increasing uniformity in independence levels among survivors.

The cumulative findings of the functional outcome and LOI are reflected in Table 1.

**TABLE 1:** Overall mean score of functional outcome and level of independence among the survivors of cardiac arrests.

**Duration of CPR**	**Immediate post-CPR (favorable/unfavorable)**		**Day 30 (favorable/unfavorable)**			**Day 90 (favorable/unfavorable) *n* = 121**	

Survivors	80		53			50	
≤ 5 min	5/16		12/2			12/0	
6–10 min	0/21		7/6			7/1	
11–20 min	1/36		15/9			15/3	
> 21 min	0/42		11/18			11/1	

**Outcome scores**	**Functional outcome**			**Level of independence**			
**Observation period**	**Mean**	**Standard deviation**	**IQR**	**Survivors (*ƒ*)**	**Mean**	**Standard deviation**	**IQR**

Immediate post‐CPR	3.72	1.04	3–5	80	2.53	2.28	0–5
Day 30	3.48	1.85	1–5	53	3.81	1.71	3–5
Day 90	3.48	1.91	1–5	50	4.3	1.5	4–5

### 4.7. Section 4: Functional Outcome Dimensions Categorized as Favorable and Unfavorable According to Duration of CPR Among CA Patients

Based on the findings in Table 2, in the immediate post‐CPR period, favorable mean scores were observed only in patients resuscitated for ≤ 5 min and 11–20 min, while no favorable outcomes were recorded in the 6–10‐min and > 21‐min groups. Unfavorable mean scores remained consistently high across all duration groups immediately after CPR. By Day 30 and Day 90, favorable mean scores improved and stabilized around 1.00–1.16 across all CPR duration groups, indicating neurological recovery among survivors. However, unfavorable mean scores remained higher (approximately 4.50–5.00), particularly in patients with prolonged CPR duration, suggesting that longer resuscitation time was associated with poorer functional outcomes.

**TABLE 2:** Comparison of the mean score of functional outcome and CPR duration among patients with cardiac arrest.

Duration of CPR	Mean score in immediate post‐CPR		Mean score on Day 30		Mean score on Day 90	
	Favorable	Unfavorable	Favorable	Unfavorable	Favorable	Unfavorable
≤ 5 min	1.4	3.87	1.00	4.50	1.00	5.00
6–10 min	—	3.85	1.16	4.86	1.00	4.92
11–20 min	2.00	3.83	1.06	4.86	1.06	4.86
> 21 min	—	3.78	1.09	4.83	1.00	4.96

### 4.8. Section 4: LOI Categorized as Dependent, Partially Dependent, and Independent According to Duration of CPR Among CA Patients

Based on Table 3, during the immediate post‐CPR, dependent mean scores were higher among patients with a shorter CPR duration (≤ 5 min), suggesting a critical threshold around 5–6 min and progressively lower with prolonged CPR duration. Partially independent mean scores were observed mainly in the ≤ 5‐min and 11–20‐min groups. By Day 30, dependency mean scores reduced considerably across most duration groups, while partially independent mean scores increased, indicating functional improvement. A slight increase in the dependency mean score (1.00) was observed in the > 20 min group. On Day 90, dependency mean scores reduced to zero across all duration groups, and partially independent mean scores further increased (ranging from 4.71 to 4.83), demonstrating sustained functional recovery irrespective of CPR duration, although better trends were still noted among those with shorter resuscitation times.

**TABLE 3:** Comparison of the CPR duration to the mean score of level of independence among patients with cardiac arrest.

Duration of CPR	Mean score in immediate post‐CPR *n* = 80			Mean score on Day 30 *n* = 53			Mean score on Day 90 *n* = 50		
	Dependent	Partially independent	Independent	Dependent	Partially independent	Independent	Dependent	Partially independent	Independent
≤ 5 min	1.00	4.00	—	0	4.50	—	0	4.83	—
6–10 min	0.84	—	—	0	4.14	—	0	4.71	—
11–20 min	0.30	4.00	—	0.50	4.50	—	0	4.73	—
> 20 min	0.18	3.00	—	1.00	4.72	—	0	4.81	—

**FIGURE 5:**
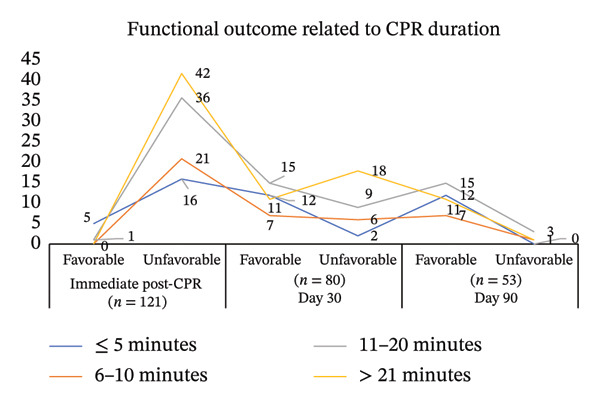
Functional outcome based on CPC related to the CPR duration during immediate post‐CPR, Day 30, and Day 90.

The study findings from Figure 5 highliight that CPR duration is a powerful predictor: of both survival and functional outcome. Shorter CPR times are overwhelmingly associated with better chances of both surviving and having a good neurological recovery. While high mortality is the dominant factor among patients with prolonged CPR and unfavorable outcomes from the later cohorts. The recovery plateaus early observed through the lens of neurological recovery for survivors are largely complete by Day 30. Patients who have a favorable outcome in one month will almost certainly maintain it at 3 months. The prognostication is explained through prognosis especially among the CPR duration of > 21 min of CPR and has an unfavorable outcome immediately post‐CPR, which has a very high probability of death, with only a small chance (11/42 ≈ 26% in these data) of surviving with a favorable outcome.

### 4.9. Inferential Analysis

The analysis demonstrated a significant relationship between the CPR duration, functional outcome, and LOI among patients with CA.

### 4.10. Functional Outcome

Repeated‐measures ANOVA showed a significant improvement in functional outcomes over time, with mean scores decreasing from 1.95 immediately post‐CPR to 1.63 by Day 30 and stabilizing at 1.62 by Day 90. This indicates that most neurological recovery occurred within the first month after resuscitation, with minimal additional improvement thereafter.

Correlation analysis revealed that the CPR duration was a significant predictor immediately after CPR (*R* = 0.140, *p* = 0.0012) but became a weak yet statistically significant predictor by Day 90 (*R* = 0.192, *p* = 0.035), suggesting that prolonged CPR has a long‐term impact on functional outcome.

The multivariate analysis assessed the effects of observation period and duration of CPR on patient outcomes.

#### 4.10.1. Effect of Observation Period

There was a highly significant effect of time on patient outcomes (Wilks’ lambda = 0.690, *F* (2,116) = 26.018, *p* < 0.001). The partial eta squared (ηp^2^ = 0.310) indicates a moderate to large effect size, meaning that approximately 31% of the variance in outcomes is explained by changes across the observation periods. This confirms a significant improvement in patient outcomes over time.

#### 4.10.2. Effect of Duration of CPR

Duration of CPR also demonstrated a statistically significant effect (Wilks’ lambda = 0.950, *F* (6,232) = 1.004, *p* < 0.001). The partial eta squared value (ηp^2^ = 0.250) indicates a moderate effect size, suggesting that about 25% of the variance in outcomes is attributable to CPR duration across time points.

### 4.11. Overall Interpretation

The findings indicate that both observation period (time) and duration of CPR significantly influence patient outcomes. While recovery over time plays a major role in improving outcomes, CPR duration also has a meaningful impact, contributing substantially to variations in functional recovery among CA patients.

### 4.12. LOI

There was a strong and statistically significant improvement in independence over the 90‐day period. The mean independence score increased markedly from 1.22 immediately post‐CPR to 4.22 by Day 90, reflecting substantial recovery. A strong negative correlation (*r* = −0.534, *p* < 0.05) immediately after CPR indicated that a longer CPR duration was associated with poorer short‐term independence. However, this association weakened and became nonsignificant by Day 30 and Day 90, suggesting that the initial effect of the CPR duration diminishes over time as other recovery factors intervene.

Multivariate analysis (Wilks’ lambda Λ = 0.312, *p* < 0.001, ηp^2^ = 0.310) confirmed a significant time effect on independence scores. Pairwise comparisons showed significant improvement from immediate post‐CPR to both Day 30 and Day 90 (*p* < 0.001), but no significant difference between Day 30 and Day 90, indicating stabilization after the first month. A very strong positive correlation between Day 30 and Day 90 independence scores (*r* = 0.935, *p* < 0.001) demonstrated that one‐month status strongly predicts three‐month outcomes.

One‐way repeated‐measures ANOVA showed that the CPR duration significantly influenced outcomes immediately after resuscitation (*F*(3,46) = 9.489, *p* < 0.001), but not at Day 30 (*p* = 0.535) or Day 90 (*p* = 0.370). This suggests that the CPR duration primarily affects early outcomes, while long‐term recovery depends on additional clinical and rehabilitative factors.

### 4.13. Interitem Correlation (Independence Measures)

Factor analysis revealed significant interitem correlations (*r* = 0.50–0.80) among independence domains such as bathing, dressing, toileting, continence, mobility, and self‐feeding. Continence showed a strong association with mobility and self‐feeding. Despite these interrelationships, communality values confirmed that each domain measured a distinct component of functional independence.

### 4.14. Section 5: Association With Selected Variables

The findings of the functional outcome suggest that while some clinical variables (initial rhythm and shockable rhythm type) significantly influenced functional outcomes, nonshockable rhythm type and defibrillation were associated with outcomes at later stages (Day 30 and Day 90). Life‐saving drug administration consistently demonstrated a significant association with functional outcome across all observation periods, highlighting its importance in both immediate and long‐term recovery among CA patients.

Chi‐square and Fisher’s exact tests conducted among Day 90 survivors (*n* = 50) revealed significant associations between LOI and marital status (*p* = 0.036), comorbid status (*p* = 0.004), and administration of life‐saving drugs (*p* = 0.036). These findings indicate that both sociodemographic and clinical variables significantly influence long‐term independence. Regression analysis further identified ROSC and duration of CPR as strong predictors of LOI.

Overall, the study concludes that the CPR duration significantly impacts immediate outcomes, but long‐term functional recovery and independence are influenced by multiple interacting clinical and demographic factors, with most recovery occurring within the first 30 days postresuscitation.

## 5. Discussion of the Findings of the Study

### 5.1. Description of Demographic Variables

**FIGURE 6:**
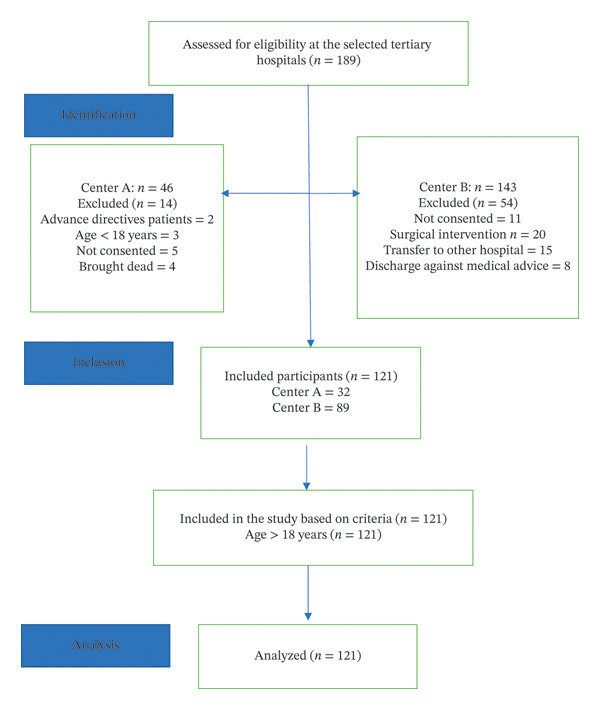
STROBE guidelines for observation study.

Based on the STROBE reporting system for observational studies as shown in Figure 6, the flowchart represents the number of patients selected in the study. The total samples for the study selected from the two tertiary care centers A and B were 46 and 111, respectively. The total number of patients selected from both the centers after excluding based on criteria as mentioned in flowchart was 121.

Based on the gender majority of patients, 89 (73.6%) were male patients, which is supported by a study that shows the 63.1% of male patients with CA further emphasizing the high risk of CA among the male population [17]. Cardiovascular disease (CVD) incidence and prevalence demonstrate a higher susceptibility among the male gender. The incidence and prevalence of CVD demonstrate a higher susceptibility among males. It has been reported that out of every 1000 individuals, six males are diagnosed with CVD compared to three females [4]. These findings highlight that, among nonmodifiable risk factors, male gender is associated with a greater susceptibility to CA.

Advancing age is associated with an increased frequency of CA, consistent with studies indicating that aging is a major risk factor for NCDs, particularly CVDs such as CA and sudden cardiac death [18, 19]. In the present study, the mean and median ages were 62.49 years and 65 years, respectively, with 60% of patients aged above 50 years. These findings align with previous research reporting a median age of 74 years among CA patients [20]. However, CA can also occur in younger individuals, with approximately 30% of cases reported in those under 45 years [21].

A study by Wiberg et al. examined the trends in survival following IHCA and rates of hospital discharge across different age groups. The findings showed that elderly individuals had the lowest survival rate to discharge (18%), with survival declining as age increased. However, the study also reported a significant improvement in survival to discharge across all age groups between 2000 and 2016 [22]. Although several factors influence outcomes, age remains an important determinant of survival.

The study findings showed that 95.8% of patients who experienced CA were married. This observation is supported by previous research, indicating increased vulnerability to cardiac events among individuals in married or disrupted marital relationships [23]. Additionally, a high incidence of CA (91.9%) was observed among urban residents, which aligns with studies suggesting an increased risk of CA associated with urbanization [24]. These findings may reflect the influence of lifestyle changes, cultural shifts, and epidemiological transition associated with urban living, contributing to the rising burden of cardiovascular mortality.

Furthermore, 32.3% of CA cases occurred in hospital wards, emphasizing that patients remain at risk at all times. However, patients who develop CA in closely monitored areas such as the ICU, HDU, cath lab, and emergency department may benefit from prompt recognition and immediate intervention, potentially reducing complications.

CA, the role of bystanders, and recovery outcomes have been extensively studied in OHCA, whereas IHCA and its underlying causes remain comparatively under‐researched [25]. A study from Denmark comparing IHCA and OHCA reported a 3:1 higher incidence of IHCA, with more favorable outcomes and noted that 62% of arrests occurred in hospital wards [25]. For the overall group, the rate of ROSC and 30‐day survival were 55.3% and 43.8%, respectively. These findings are comparable to a Swedish study reporting 63% ROSC and 30% survival at 30 days [26] In contrast, the Denmark study reported ROSC and 30‐day survival rates of 49% and 27% for IHCA, and 24% and 17% for OHCA, respectively [25]. These results suggest relatively lower survival probabilities at 30 days following IHCA and even poorer outcomes for OHCA.

Although many studies report ROSC rates, they often lack specificity regarding the time component of ROSC. Sustained ROSC, defined as the maintenance of a stable cardiac rhythm for at least 20 min following resuscitation, is considered an important indicator when making ToR decisions and is associated with improved functional outcomes among survivors. Therefore, integrating the concept of sustained ROSC into resuscitation decision‐making may enhance clinical management during CPR. [27].

### 5.2. Description of Duration of CPR Among CA Patients

The study findings revealed a mean CPR duration of 18.97 ± 14.7 min and a median duration of 15 min (IQR: 10–26 min), with 65.3% of patients receiving resuscitation for more than 10 min. Previous studies have reported similar median CPR durations ranging from 17 to 25 min, with survival decreasing as the CPR duration increases [28–31]. In the present study, 34.7% mortality at Day 90 was observed among patients who underwent resuscitation for more than 20 min. These findings support recommendations that resuscitation efforts should continue for at least 20 min before considering the termination of CPR.

However, the AHA guideline recommending the termination of CPR at 6 min under specific criteria—unwitnessed arrest, no ROSC, and no shock delivered in OHCA—was not supported by the present study findings. Similarly, a systematic review of 92 IHCA studies reported low‐certainty evidence supporting the UN10 rule (unwitnessed arrest and no ROSC within 10 min for an initial non‐shockable rhythm), with no definitive recommendations for the termination of CPR in IHCA [32].

The study demonstrated a significant improvement in functional outcomes and LOI among CA patients over the 90‐day observation period. Repeated‐measures ANOVA showed a progressive reduction in functional outcome scores from 1.95 immediately post‐CPR to 1.63 at Day 30 and 1.62 at Day 90, indicating that most neurological recovery occurred within the first month after resuscitation, with minimal improvement thereafter. Time was a major determinant of outcome (Wilks’ lambda = 0.690, *p* < 0.001, ηp^2^ = 0.310), explaining approximately 31% of outcome variance, while the CPR duration also significantly influenced outcomes (Wilks’ lambda = 0.950, *p* < 0.001, ηp^2^ = 0.250). Correlation analysis further showed that the CPR duration was a significant predictor immediately after resuscitation, although its influence weakened over time, suggesting that early outcome is more sensitive to resuscitation duration.

A systematic review encompassing 39 studies reported that 78.9% of patients who experienced CA had poor neurological outcomes, while 9.9% of survivors exhibited significant neurological impairment. Psychological and physical morbidities were also frequently observed, with anxiety affecting 13.1% of survivors and reported pain or discomfort in 60.5%. However, the synthesis was constrained by substantial heterogeneity in outcome measurements and a high risk of bias across most included studies [33].

Another systematic review that included 20 studies reported that survival following CA ranged from 11.8% to 39.5%. Survivors demonstrated an elevated risk of impaired cerebral function and discharge to institutional care. Although one study reported no significant difference in postresuscitation quality of life, another observed an increased requirement for assistance with ADL among survivors [34]. In addition, a prospective SURVIVE‐ARREST study involving 368 patients with IHCA demonstrated a 30‐day survival rate of 53.3%. Among survivors, 72.9% achieved favorable neurological outcomes, which were significantly associated with ROSC without mechanical circulatory support, receipt of coronary angiography or percutaneous coronary intervention, and administration of antibiotic therapy [35].

Improved favorable outcomes have been reported among patients resuscitated with extracorporeal CPR (ECPR), which is increasingly studied as a prognostic approach for assessing functional recovery in CA survivors. Research primarily focuses on prearrest determinants such as younger age, baseline cardiac function, laboratory indicators, and duration of no‐flow or low‐flow arrest periods [36–39]. Despite its potential benefit, ECPR remains underutilized, particularly in India, with limited empirical evidence supporting its routine clinical application.

Prognostic tools have also been developed to guide clinical decision‐making. The resuscitation predictor tool by Cooper et al. estimates only a 3.8% probability of 24‐h survival among patients older than 70 years with primary CA and initial normal sinus rhythm [40]. Multimodal assessment approaches, including infrared pupillometry, echocardiographic detection of cardiac standstill, and end‐tidal carbon dioxide (ETCO_2_) levels below 10 mmHg after 20 min of resuscitation as recommended by the AHA, may assist in ToR and prognostication. Additionally, scoring systems such as the GO‐FAR score [41, 42], Clinical Frailty Scale [43, 44], and GOTO algorithm for OHCA [45, 46] support DNR‐related decisions, although no definitive time threshold for TOR is currently recommended. [40]. The above studies highlight on the need for unification on multiple factors such as resuscitation duration, sustained ROSC [27], criteria for ToR, and clinical parameters to evaluate the clinical outcome among CPR patients. Multiple studies have assessed functional outcomes after CA, but variability in outcome measures persists. This issue has been highlighted by the International Liaison Committee on Resuscitation, which recommends developing a Core Outcome Set for Cardiac Arrest (COSCA) for standardized outcome measurement [47, 48]. While no COSCA studies have been conducted in India, this study is notable for assessing outcomes using two scales consistent with the recommendations of the International Liaison Committee on Resuscitation.

## 6. Description of LOI Among CA Patients

The review literature identifies only one study measuring the outcome of the CA patient based on LOI and showed 63.3% of patients independent at the time of discharge [49]. A conclusive validation cannot be based on a single study, and more large‐scale studies must be duplicated to identify the correlation of LOI among the CA patients.

### 6.1. Comparison Between CPR Duration and LOI in Terms of Dependency and State of Independency Among CA Patients

Evidence on the LOI among CA survivors is limited, with only one study reporting that 63.3% of patients were independent at discharge. Due to the small sample size and lack of repeated large‐scale studies, definitive conclusions regarding independent outcomes cannot be drawn. Similarly, comparisons between CPR duration and independence status remain inconclusive, as existing research has not performed detailed statistical correlation analyses, and no supporting or contradictory studies are available. There are numerous studies with LOI frequently assessed among heart failure and chronic diseases, while there is only one study done among the CA patients. Recommendations cannot be made based on the single study with the sample size in the study is less than 200 patients[49].

Overall, the assessment of quality of life and independence after CA is a novelty of this study. The literature review highlights only one study done in Australia by researchers Pound et al., which studied about the LOI among the CA patients and did not analyze the correlation as a statistical test [49]. Hence, no comparable or contradictory evidence is available to validate the present findings.

Future research should integrate holistic and nursing‐centered outcome measures, including the qualitative evaluation of patient and family transitions, and strengthen evidence to support resuscitation outcome assessment.

The assessment of QOL represents a major novelty of this study and aligns with the growing emphasis on core outcome sets for evaluating resuscitation effectiveness. Previous research has primarily focused on clinically measurable outcomes, while patient, family, and caregiving transition experiences have received less attention. From a holistic care perspective, the integration of nursing theory into CA management is essential to address these transitional dimensions of recovery and patient support. Strengthening nursing curriculum orientation is recommended to enhance future clinical practice [50] Furthermore, additional qualitative studies are warranted to better explore and measure QOL outcomes among CA survivors.

### 6.2. Major Findings of the Study

**FIGURE 7:**
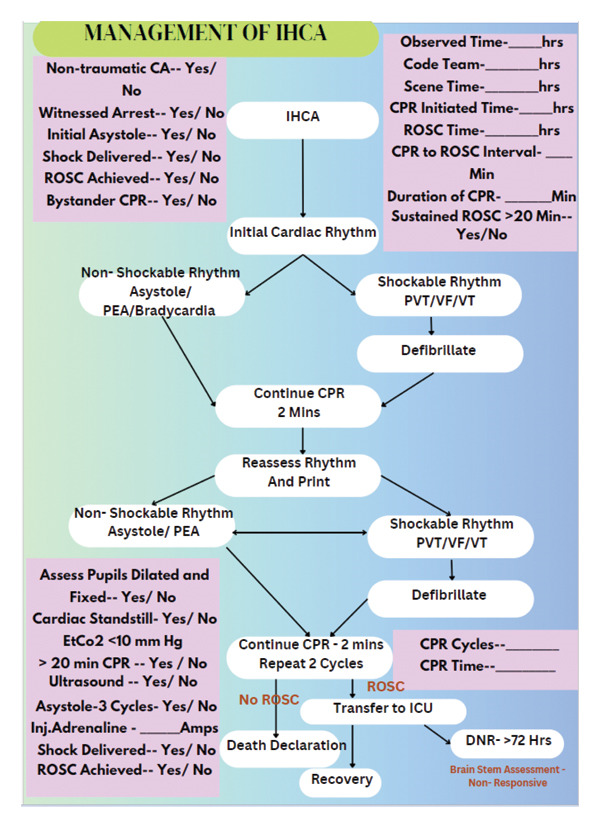
ADAM’S algorithm for ToR in IHCA.

The major findings of the study highlight the incidence of IHCA increases with age and cardiac origin is the dominating cause of the CA predominately among the male gender. Based on the study, the individuals from urban are more at risk of developing the CA most probably due to the underlying CVDs, which lie dormant and incidentally identified through an incident of IHCA. Major percentage of the patients were resuscitated for minimum 20–25 min irrespective of the cause and initial rhythm. The majority of the patients presented with initial cardiac NSR specifically asystole. The immediate survival in the post‐CPR phase was 66.2%, which reduced to 43.9% by Day 30. In conclusion, the overall survival rate by Day 90 to be 41.3%, which is considered good outcome in comparison with many research studies. And the relevant explanation to the increased survival is that the majority of the patients had curable cardiac diagnosis as the cause of the CA. The LOI also demonstrated consistent improvement of the patient from the state of dependency in the immediate post‐CPR phase to Day 90 and were at the optimal level of the functional state with minimum dependence. The study findings also demonstrated a strong correlation to initial rhythm specifically SR, life‐saving drugs, duration of CPR, and ROSC, which was also identified as the strongest predictors for the meaningful outcome among the patients. The major study findings recommend an algorithm that can be further investigated toward clinical decision‐making related to the ToR, which is viewed in Figure 7—Anandhi Deva Amirtharaj’s (ADAM’S) algorithm for ToR in IHCA.

### 6.3. Implications

Nursing practice should be guided by evidence‐based guidelines to ensure high‐quality, continuous CA care. Current evidence suggests that prolonged CPR without sustained ROSC warrants careful consideration of termination protocols and rhythm‐based management. Adherence to AHA guidelines, supported by regular BLS and ACLS certification, is essential for effective resuscitation. Early deterioration detection tools such as the Adult Early Warning Score can facilitate timely intervention in high‐risk patients. Nurse involvement in research to establish core outcome measures for post‐CA independence should be encouraged, as this area remains underexplored.

Nursing administration should adopt research‐informed standards of care, including the implementation of the Utstein template for accurate CA documentation and promotion of prospective outcome studies. Institutional leaders should emphasize continuing education, protocol evaluation, and system‐level preparedness across prevention, resuscitation, and recovery phases. Development and enforcement of ToR policies are also necessary for system‐wide implementation.

Nursing education must adapt to evolving clinical challenges by incorporating research‐based curricula, simulation training, and competency‐focused learning strategies. Updated educational content should include emerging CA phenotypes, in‐hospital and out‐of‐hospital resuscitation management, and contemporary outcome assessment tools. Continuous curriculum refinement and integration of current evidence are essential to prepare nurses for advanced clinical practice and improve patient outcomes.

The growing complexity of CA management highlights the critical need for nursing research to strengthen evidence‐based practice in resuscitation care, functional recovery, and quality of life outcomes. Nursing research has traditionally focused on areas such as quality of life, clinical management, and nurse burnout. However, there remains a dearth of nursing‐led research on CPR, patient assessment, and outcome evaluation, partly due to perceived limitations in research topic selection within the nursing profession. Expanding the scope of nursing research to include resuscitation and outcome‐based studies is essential for advancing nursing science and promoting evidence‐informed practice.

There remains limited research focusing on patient independence, long‐term functional recovery, and holistic postresuscitation transitions, indicating a significant knowledge gap in nursing science. Nursing research should be broadened to include more outcome‐oriented studies, which will strengthen advanced nursing practice and support clinical innovation. Alignment with evidence‐based resuscitation recommendations, including core outcome standardization advocated by the International Liaison Committee on Resuscitation, further underscores the importance of nursing‐led research in developing reliable outcome measurement frameworks.

Nursing research is essential to evaluate optimal CPR duration thresholds, predictors of sustained ROSC, and factors influencing neurological and functional recovery. Additionally, studies exploring patient, family, and caregiver transition experiences are necessary to integrate holistic care perspectives into CA management. Research is also required to validate early warning systems, ToR protocols, and independence‐based outcome scales within diverse clinical settings. Strengthening nursing research in these areas will support curriculum development, improve clinical decision‐making, and enhance survival and quality of life outcomes among CA patients.

### 6.4. Recommendations

The following recommendations were made from the present study for further research.

Comparison studies can be done among IHCA and OHCA patients related to CPR techniques, sustained ROSC, depth of chest compressions, and bystander resistance in initiating CPR. A similar study can be duplicated specifically among Medical ICU or a more homogenous group of patients. ADAM’s algorithm generated as the hypothesis and conceptual framework must be evaluated for statistical significance toward ToR in terms of a positive predictive value. And more research can be done on the LOI among CA patients.

### 6.5. Limitations

The limitations of the study were that the study was conducted in tertiary care hospitals and majority of the patients were dominated by cardiac patients. Nontraumatic multifactorial CA would have provided a good insight based on the heterogenicity of the patients to correlate between the multifactorial diagnosis and the influence of meaningful outcome. The pandemic COVID‐19 was a major limitation in the study due to the rapid decrease in the acuity of the patients in PIMS, which was a good setting for the study. Review of the literature highlighted that this concept was usually studied as nationwide to study about the improvement in the healthcare delivery system and assess the mortality rates over the decade. Hence, the small size of the study decreased the magnitude and impact of the study in terms of representation of the country. The average number of days from admission to CA was not assessed in the study.

## 7. Conclusion

In conclusion, the traditional recommendation to continue resuscitation for a minimum of 20 min irrespective of initial rhythm requires reconsideration. Continuation of CPR should be guided by multimodal prognostic evaluation rather than adherence to a fixed duration threshold. The clinical dilemma of “how long is long enough?” remains central to the ToR decisions, supporting individualized assessment based on physiological, neurological, and hemodynamic indicators. Evidence and guideline perspectives from the International Liaison Committee on Resuscitation favor integrated prognostic strategies over rigid time‐based protocols.

Unwitnessed CA, absence of sustained ROSC, and failure to deliver shock therapy may be considered indicators when evaluating the ToR. Functional outcome and LOI showed marked improvement by Day 30 with minimal additional change by Day 90, suggesting that meaningful prognosis is most evident within the first month after resuscitation. Therefore, decisions regarding withdrawal or ToR should be approached cautiously during the early recovery period, given the substantial potential for clinical improvement.

## Funding

This is not a funded study.

## Conflicts of Interest

The author declares no conflicts of interest.

## Data Availability

The data that support the findings of this study are available on request from the corresponding author. The data are not publicly available due to privacy or ethical restrictions.
